# Modeling gene-by-environment interaction in comorbid depression with alcohol use disorders via an integrated bioinformatics approach

**DOI:** 10.1186/1756-0381-1-2

**Published:** 2008-07-17

**Authors:** Richard C McEachin, Benjamin J Keller, Erika FH Saunders, Melvin G McInnis

**Affiliations:** 1 Department of Psychiatry, University of Michigan, Ann Arbor, Michigan, USA; 2 National Center for Integrative Biomedical Informatics, University of Michigan, Ann Arbor, Michigan, USA; 3 Department of Computer Science, Eastern Michigan University, Ypsilanti, Michigan, USA

## Abstract

**Background:**

Comorbidity of Major Depressive Disorder (depression) and Alcohol Use Disorders (AUD) is well documented. Depression, AUD, and the comorbidity of depression with AUD show evidence of genetic and environmental influences on susceptibility. We used an integrated bioinformatics approach, mining available data in multiple databases, to develop and refine a model of gene-by-environment interaction consistent with this comorbidity.

**Methods:**

We established the validity of a genetic model via queries against NCBI databases, identifying and validating TNF (Tumor Necrosis Factor) and MTHFR (Methylenetetrahydrofolate Reductase) as candidate genes. We used the PDG-ACE algorithm (Prioritizing Disease Genes by Analysis of Common Elements) to show that TNF and MTHFR share significant commonality and that this commonality is consistent with a response to environmental exposure to ethanol. Finally, we used MetaCore from GeneGo, Inc. to model a gene-by-environment interaction consistent with the data.

**Results:**

TNF Alpha Converting Enzyme (TACE) activity is suppressed by ethanol exposure, resulting in reduced TNF signaling. TNF binds to TNF receptors, initiating signal transduction pathways that activate MTHFR expression. MTHFR is an essential enzyme in folate metabolism and reduced folate levels are associated with both AUD and depression. Integrating these pieces of information our model shows how excessive alcohol use would be expected to lead to reduced TNF signaling, reduced MTHFR expression, and increased susceptibility to depression.

**Conclusion:**

The proposed model provides a novel hypothesis on the genetic etiology of comorbid depression with AUD, consistent with established clinical and biochemical data. This analysis also provides an example of how an integrated bioinformatics approach can maximize the use of available biomedical data to improve our understanding of complex disease.

## Background

Comorbidity of Major Depressive Disorder (depression) with Alcohol Use Disorders (AUD) is well documented [1]. Further, significant statistical association between depression and alcohol dependence has long been established [2]. For depressed individuals, the odds ratio for alcohol dependence may be as high 4 to 7, relative to controls, with a similar increase in prevalence of depression among alcohol dependent individuals [3,4]. This association between depression and AUD suggests that there may be one or more common underlying elements influencing susceptibility to both disorders.

Depression, AUD, and the comorbidity of depression with AUD are complex traits, demonstrating both genetic and environmental influences on susceptibility. Depression is familial, with estimates of heritability as high as 54% [5]. AUD is also familial, with heritability estimates in the range of 50 to 60% [6]. Comorbidity of depression with AUD also shows evidence of both genetic and shared environmental influences [7-10]. Given evidence of association between depression and AUD, as well as evidence that both the individual traits and the comorbidity are influenced by both genetic and environmental factors, we used an integrated bioinformatics approach, maximizing the use of available data in multiple databases, to investigate the hypothesis that interacting genetic and environmental influences are common underlying elements of susceptibility to comorbid depression with AUD. We report the development and testing of a model of this comorbidity, as well as implications for future research into comorbid depression with AUD.

## Methods

### Queries against NCBI Databases

In this analysis, we used available data in NCBI databases, plus three software suites (DAVID, PDG-ACE, and GeneGo). To identify potential candidate genes, we queried the Entrez Gene database [11] for "depression AND ethanol AND human [orgn]". Since Entrez Gene surveys the whole human genome, this approach is complementary to Whole Genome Association (WGA) analysis [12-14]. WGA surveys many genetic variants (e.g. ~1 million Single Nucleotide Polymorphisms (SNPs)) across the genome, while this text-based query surveys all genes in Entrez Gene. WGA provides indirect information on potential associations of genes with disease by associating nearby SNP variants with disease, while Entrez Gene queries associate genes with disease based on curated text describing the genes. Each approach has advantages and disadvantages, highlighted in Table 1, though we believe that the query-based approach offers significant advantages in the initial phase of candidate gene analysis. First, in recent years Entrez Gene has become a rich source of information on many genes, while WGA typically focuses on a very limited information source (e.g. SNP association). Second, data provided by NCBI are free to the users. While some of the genotypic and phenotypic data required for WGA are becoming available in free public repositories, these data are generally expensive to acquire. Third, data derived from curated sources such as Entrez Gene are backed by multiple rounds of experimentation and peer review, so the believability of a positive result, as measured by Positive Predictive Value [PPV = True Positives/(True positives + False Positives)], is very high. Given these strengths, a query based approach should be considered for the initial phase of candidate gene analysis, which will then maximize the value of subsequent association testing.

**Table 1:** Comparison of Whole Genome Association (WGA) to text based, Entrez Gene, search for candidate genes.

	**Identifying Candidate Genes**	
	**WGA**	**Text Based Search**

**Data Source**	Genotyping and Phenotyping	Entrez Gene
**Current Availability of Data**	Limited	Available
**Expense**	High	Free (Taxpayer Subsidized)
**Sensitivity**	Low; limited number of markers tested	Low; much remains unknown about most genes
**Positive Predictive Value**	Low; many false positives	High; curated information is backed by experimental data and peer review
**Population Specific**	Yes	Generally no, although population specific information may be included in some records
**Specific Variants Identified**	Yes, although SNPs that tag haplotypes may not be disease causing variants	Generally no, although specific variants may be annotated. Separate genotyping is usually required for validated candidates
**Multi-Gene Effects on Phenotype**	Limited	Yes
**Complex Phenotypes**	Limited	Yes

Text based searches, using data at NCBI as the first source for candidate gene data, provide advantages in data availability, price, and positive predictive value.

The Entrez Gene database provides reliable information about most genes, but the Entrez Gene record is a summary, so the information it provides is necessarily limited. Therefore, we queried a second NCBI database, PubMed [15], for more complete information on a variety of hypotheses developed in this analysis. Table 2 shows the short form of each PubMed query in column 1 and the citation count is in column 2. For each PubMed query, the actual query was qualified by Medical Subject Headings (MeSH) annotation [16] and the detailed queries are included in Additional file 1. Publications are annotated by MeSH terminology to provide a consistent way to retrieve information in publications that may use different terminology for the same concepts. In addition, querying PubMed by MeSH annotation yields a high likelihood that publications returned by the query refer to the queried terms in the appropriate context (high PPV). For example, in looking for association between depression and AUD, we queried PubMed for "("Depressive Disorder" [Mesh] OR "Depression" [Mesh] OR "Depressive Disorder, Major" [Mesh]) AND ("Ethanol" [Mesh] OR "Alcohol-Induced Disorders, Nervous System" [Mesh])". The resulting 263 citations returned are consistent with the statistically significant association between depression and AUD found by Hasin and Glick [2]. Using the University of Michigan's electronic journals library, we made an exhaustive review the 263 citations returned by this query to quantify the PPV of this MeSH qualified PubMed query. In this review, we looked for clear evidence that the authors were studying AUD and depression, in English language publications, either full text or abstracts. We were able to unequivocally assess the accuracy of MeSH annotation for AUD and depression in 179 citations. Of these 179 publications, 171 clearly refer to both AUD and depression, though not necessarily the comorbidity of AUD with depression. Of the eight remaining publications, five are correctly annotated for ethanol, but ethanol is used as an important reagent in the experimental procedures. The final three publications are incorrectly annotated for ethanol, though they refer to pharmacologically related chemicals (flupenthixol, haloperidol, and pirbuterol). We consider these eight publications to be false positives with respect to our original query, yielding a 95.5% (171/179) PPV for our query.

**Table 2:** PubMed queries and citation counts

**Short form, PubMed Queries**	**PubMed Citation Count**
depression AND ethanol	263
depression AND MTHFR	12
ethanol AND MTHFR	5
depression AND ethanol AND MTHFR	0
depression AND TNF	92
ethanol AND TNF	321
depression AND ethanol AND TNF	1
depression AND NFkB	2
ethanol AND NFkB	185
depression AND ethanol AND NFkB	0
depression AND TNFR	19
ethanol AND TNFR	44
depression AND ethanol AND TNFR	0
depression and APOE	91
ethanol and APOE	42
depression AND ethanol AND APOE	0

PubMed queries were used to search for evidence consistent with various hypotheses on 9 December, 2007. Queries were qualified by Medical Subject Headings (MeSH) annotation to minimize spurious associations. The actual queries, including MeSH qualifications, are provided as Additional file 1. Column 1 has a short form of the query and column 2 shows the number of citations that meet the query criteria.

There are, however, limitations to the use of MeSH annotation in querying publications. First, while MeSH contains thousands of Medical Subject Headings, it is not an exhaustive annotation of all potential biomedical contexts. Second, there may be missing annotation or ambiguity in the annotation for some publications. For example, as mentioned in the PPV analysis, not all of the 171 papers that we counted as True Positives were specifically about comorbid AUD with depression. The earlier papers tended to refer to AUD and depression as co-occurring phenotypes, while the later papers focused on evaluating the comorbidity. There also may be ambiguity in the user's choice of MeSH terms queried, and some publications may report negative associations, though the bias against publication of negative results minimizes the number of these papers. And finally, a lack of citations is not evidence of a lack of association. While co-occurrence of queried terms is not proof of association, co-occurrence does provide a quantitative measure of peer reviewed research on the association of these specific terms and complements other forms of data that can be mined. In future analyses, Natural Language Processing will help to improve these queries by mining the full text of publications for a wide variety of concepts, and provide additional evidence of association among terms. Given believable candidate genes, derived from queries of available data at Entrez Gene and PubMed, we continued the analysis using DAVID, PDG-ACE, and MetaCore (GeneGo, Inc.) to understand how these candidate genes may be related to the etiology of AUD, depression, and comorbid AUD with depression.

### DAVID, PDG-ACE, and GeneGo

DAVID software, the PDG-ACE algorithm, and the GeneGo suite perform related tasks. The essential assumption in the use of all three tools is that, in complex diseases, multiple genetic influences converge on a single phenotype. The three tools use different approaches to identify how multiple genes may influence the phenotype of interest and assess the statistical significance of the results seen.

DAVID [17] uses a database of available gene annotation to overlay candidate genes onto predefined gene sets. For example, genes may be organized into predefined sets based on annotation for their participation in a Gene Ontology function, process, or component [18]. A set of candidate genes is then derived from a genotype/phenotype analysis (e.g. WGA, microarray expression, or text-based database query) and the candidate genes are overlaid on the predefined gene sets. If a large proportion of the candidate genes are found to be annotated for a given gene set (e.g. an over-represented Gene Ontology process), the candidate genes may have an important impact on the process represented by that gene set. Since the candidate genes were initially derived from a genotype/phenotype relationship, the over-represented process may be important in the phenotype [19].

However, since much gene annotation remains incomplete, PDG-ACE [20] [see Additional file 2] serves as an adjunct to software that depends on gene annotation. As noted, the Entrez Gene database offers a reliable summary of information on a wide range of genetic and environmental influences for each gene (e.g. gene function, disease influences, localization, environmental effects). However, much of the information available in Entrez Gene is free text, rather than formal annotation, limiting the use of tools like DAVID. We created the PDG-ACE algorithm to overcome this limitation by mining Entrez Gene text to identify biomedical keywords that are common and significantly over-represented in the descriptions of genes at a selected locus pair, relative to all locus pairs in the genome.

The PDG-ACE algorithm uses a controlled vocabulary of biomedical keywords to limit the search to concepts likely to be useful in understanding disease etiology and show statistically significant over-representation. For a given locus pair, PDG-ACE mines the Entrez Gene text to find keywords from the controlled vocabulary that are common across the locus pair. For keywords that are common across the locus pair, significance of over-representation is established by permutation testing. Keywords that are common and significantly over-represented across the locus pair highlight rare combinations of genes that share the biomedical concepts associated with the keywords. Testing of the PDG-ACE algorithm, using both positive controls (documented gene-gene interactions in complex disease) and negative controls (randomly selected gene pairs), demonstrates that PDG-ACE provides insight into the etiology of true genetic interactions, while excluding loci where the data is insufficient to identify interactions [20]. As with DAVID, the loci are initially defined by the disease phenotype, so the over-represented keywords may offer insight into the nature of a genetic interaction in disease etiology. In applying the PDG-ACE algorithm, we define genetic interaction to mean a "statistically significant multi-gene or multi-gene-by-environment influence on the phenotype", consistent with Hartman et al. [21]. This definition is deliberately broad, allowing PDG-ACE to identify unannotated influences on the phenotype, ranging from well-defined influences (e.g. epistasis, protein-protein binding, canalization, genetic robustness, buffering) to completely novel influences, as long as the influence is multi-gene (or multi-gene-by-environment) and statistically significant. The complete list of keywords used in this analysis is included in Additional file 3. Software implementing the PDG-ACE algorithm is available on request from Dr. Ben Keller, bkeller@emich.edu.

The GeneGo suite [22] uses the MetaCore database of documented gene-gene (protein-protein) interactions [23], as well as a set of graphical and statistical tools, to allow researchers to build gene networks based on relationships among genes. The MetaCore interactive database has been manually curated from publications describing interactions among proteins and small molecules of biological relevance in humans. We used GeneGo to place our candidate genes into a cellular context, evaluate the significance of gene networks that our candidates participate in, and build a model that integrates all of the information derived in our analysis. Specifics of the parameter settings used in the GeneGo analysis are included in Table 3. Notably, we accepted only direct interactions, curated by human reviewers.

**Table 3:** Parameter settings used in GeneGo analysis

**Options**	**Setting**
Use canonical pathways	Yes
Network type	Merged
Build options	Analyze network (transcription factors)

**Advanced options**	

Discard objects user list	No
Use indirect interactions	Discard
Use interactions weights	No
Add complementary objects	Yes
Filter interactions by confidence level	Curated only
Use binding interactions (special cases)	Discard
Group networks	No
Discard objects experiments	No
Use unspecified reactions	Discard

GeneGo analysis was performed with MetaCore™ version 4.5, build 11165, on 26 November 2007. *TNF *and *MTHFR *were the input gene set.

## Results

**Figure 1:**
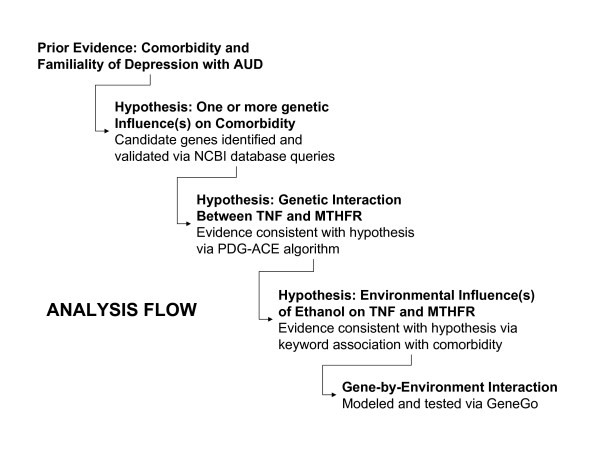
**Analysis Flow**. Analysis started with prior evidence of potential genetic and environmental influences on comorbidity, followed by testing for genetic influences, genetic interactions, and environmental influences. The resulting model of gene-by-environment interaction is consistent with clinical and biochemical data.

Based on prior evidence consistent with genetic and environmental influences on susceptibility to comorbid depression with AUD, we started the analysis with the hypothesis that genetic influences participate in depression and AUD. Figure 1 shows the analysis flow, including the initial hypothesis, three rounds of hypothesis testing and generation, and model building based on the results of our testing.

### Genetic Influences on Comorbidity

We first searched for evidence of genetic influences on depression and AUD by querying NCBI's Entrez Gene database for "depression AND ethanol AND human [orgn]". This query returned exactly three genes: Tumor Necrosis Factor (*TNF *superfamily, member 2, GeneID 7124), Methylenetetrahydrofolate Reductase (*MTHFR*, 5,10-methylenetetrahydrofolate reductase (NADPH), GeneID 4524) [24-29], and *APOE *(apolipoprotein E, GeneID 348). However, the Gene Ontology annotation for APOE, "response to ethanol", is inferred from electronic annotation, rather than manual curation. In an effort to use only the most reliable information to establish our initial candidate genes, we excluded *APOE *from the formal analysis. However, we have included a brief analysis of *APOE *in the results.

We searched PubMed for supporting information on potential roles of the proteins coded by *TNF *and *MTHFR *in depression, AUD, and comorbid depression with AUD. In searching PubMed, we found evidence that both TNF and MTHFR have been associated with depression and with ethanol in the literature, but not with the comorbidity of depression with AUD (Table 2). Notably, the result in Table 2 showing that TNF is associated with both depression and ethanol in a single publication is one of the false positive results identified in the PPV analysis. We didn't find evidence of MTHFR being associated with both depression and AUD in any single paper.

In total, the published literature is consistent with the hypothesis that *TNF *and *MTHFR *may both influence, or be influenced by, depression and AUD. We consider *TNF *and *MTHFR *to be valid candidate genes for both depression and AUD. Given these candidate genes, we next sought evidence that they participate in the comorbidity via genetic interaction.

### Interaction between TNF and MTHFR via DAVID and PDG-ACE

Given evidence of genetic influences on depression and AUD, we refined the hypothesis to include interaction between TNF and MTHFR. We first used DAVID software to explore functional annotation consistent with interaction between TNF and MTHFR. DAVID looks for over-representation of candidate genes in signaling or metabolic pathways, Gene Ontology processes, etc., based on available gene annotation. TNF and MTHFR were not found to be significantly over-represented in any gene set tested by DAVID.

Since most genes are only partially annotated, we next explored potential interactions between TNF and MTHFR via the PDG-ACE algorithm [20]. A total of 149 keywords were found to be common across the TNF/MTHFR gene pair, out of 2,531 keywords in this controlled vocabulary. We checked these 149 common keywords for over-representation using 10 million iterations in permutation testing. After a Bonferroni correction for 2,531 hypothesis tests, 27 keywords were found to be significantly over-represented (p-value < 0.05) in the TNF/MTHFR gene pair, relative to randomly selected gene pairs in the human genome. Six of these 27 keywords were found to be used in different contexts in the TNF and MTHFR Entrez Gene records and were eliminated; leaving 21 keywords that are common and significantly over-represented across the TNF/MTHFR gene pair (Table 4). Given this evidence of interaction between TNF and MTHFR in AUD and depression, and consistent with expected environmental influences on complex diseases, we further refined the hypothesis to include gene-by-environment interaction influencing the comorbidity.

**Table 4:** Significant biomedical keywords identified by PDG-ACE across the TNF/MTHFR gene pair, and relevance to depression with AUD

***Keyword***	**# PubMed citations for query:**
Ethanol	263
Consumption	29
Background	12
Intake	10
Child	9
Mortality	6
Transplant	2
Mineral	1
Hemodialysis	0
Abdominal	0
Carotid	0
Wall	0
Preoperative	0
Lumbar	0
kidney transplantation	0
Spine	0
Homozygote	0
Mediterranean	0
hormone replacement therapy	0
p53 gene	0
Sickle	0

Each keyword derived from the PDG-ACE analysis (column 1) was queried against the PubMed database via the query: ("Depressive Disorder" [Mesh] OR "Depression" [Mesh] OR "Depressive Disorder, Major" [Mesh]) AND ("Ethanol" [Mesh] OR "Alcohol-Induced Disorders, Nervous System" [Mesh]) AND *Keyword*. Citation counts are used as an indication of the keyword's relevance in comorbid depression with AUD.

### Environmental Influence of Ethanol Exposure

Each significantly over-represented keyword identified by PDG-ACE represents one hypothesis on the etiology of a genetic interaction between TNF and MTHFR in depression and AUD. We tested each of these hypotheses by querying PubMed for "depression AND ethanol AND *keyword*" (Table 4). As in the previous round of testing, we used MeSH annotation to limit the queries and we used citation counts to provide indications of relevant association in the literature. Notably, since ethanol is a keyword in the PDG-ACE controlled vocabulary that we used, it acts as a positive control in this analysis and is the top keyword when ranked by relevance. In addition to ethanol, the top keywords ranked by relevance to depression and AUD are "consumption", "background", and "intake". In the Entrez Gene records for TNF and MTHFR, intake and consumption both refer to intake of alcohol, while background refers specifically to genetic background. Results of the PDG-ACE analysis are consistent with the hypothesis that genetic background, via TNF and MTHFR, as well as environmental influences, via alcohol intake or consumption, are interacting elements of susceptibility in comorbid depression with AUD. Given evidence of genetic interaction between TNF and MTHFR, as well as gene-by-environment interaction in comorbid depression with AUD, we next sought to understand TNF and MTHFR in a cellular context.

### Model Building via GeneGo

We used the GeneGo suite [22,23] to place TNF and MTHFR in a cellular context. Results of the PDG-ACE analysis suggested that ethanol consumption exerts an environmental influence on this genetic system. Also, TNF is a secreted protein that responds to the environment by binding TNF receptors to influence intracellular signal transduction pathways that regulate gene expression. Given these inputs, we focused our GeneGo analysis on cell signaling via signal transduction and the regulation of gene expression within the cell (input parameters in Table 3).

**Figure 2:**
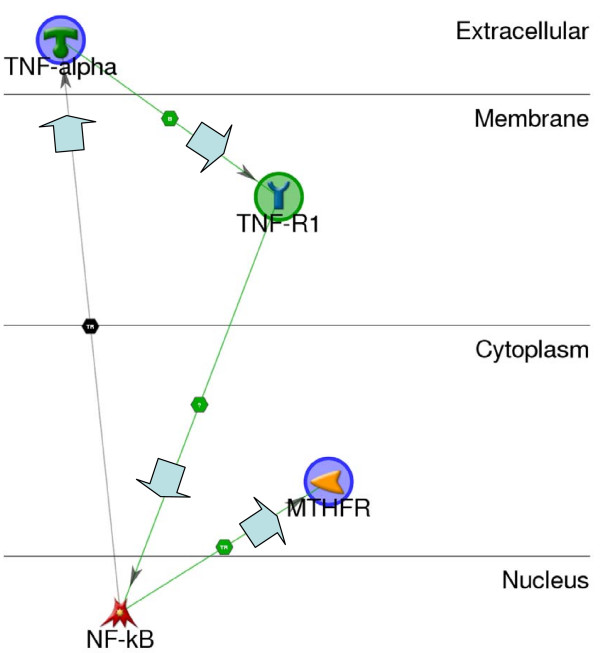
**Top scoring GeneGo network including TNF and MTHFR**. This four-gene network is the best fit found by GeneGo (p-value 3.45e-08) for TNF and MTHFR. TNF (TNF-alpha) activates TNFR (TNF-R1), which then activates NF-kB. NF-kB regulates the expression of both MTHFR and TNF, creating a feedback loop among TNF, TNFR, and NF-kB.

The most significant network found by GeneGo (Figure 2) shows a feedback loop among TNF (a.k.a. TNF-alpha), TNF-R1 (a.k.a. TNFRSF1A, GeneID 7132), and NF-kB (a.k.a. NFKB1, GeneID 4790). Note that these proteins do not act in isolation, but the GeneGo graphic shows the essential elements of the network based on annotation in GeneGo's MetaCore database. In the network identified by GeneGo, conditions in the extracellular environment are sensed inside the cell via binding of TNF to TNF-R1, activating TNF-R1 [30,31]. Activated TNF-R1 activates NF-kB [32,33], which subsequently activates the expression of both TNF [34] and MTHFR [35,36]. Given the potential for TNF-R1 and NF-kB to impact this network, we queried PubMed for potential influences of these genes on depression and AUD (Table 2). We found citations consistent with roles for both of these genes in depression and AUD.

Genetic variation or extracellular signals that affect any part of the feedback cycle (Figure 2) would tend to be amplified over a number of cycles, potentially leading to a growing imbalance in the system over time. In this network, NF-kB activates the expression of MTHFR, which metabolizes folate, so imbalances in the system would be expected to lead to imbalances in folate metabolism. This result is consistent with evidence of decreased folate levels in alcoholism [37]. In addition, reduced folate levels are associated with depression [38], where research on etiology suggests that reduced folate levels may alter neurotransmitter metabolism [39-41].

**Figure 3:**
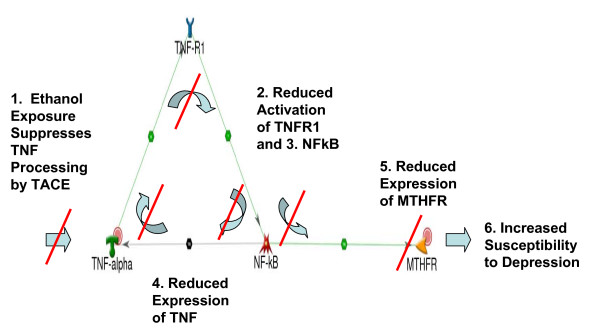
**Proposed Model of Comorbid Depression with AUD**. This model, incorporating evidence gathered in hypothesis testing, and consistent with an environmental effect of ethanol on the genetic network, shows how ethanol consumption would be expected to result in decreased folate metabolism and increased susceptibility to depression for genetically susceptible individuals.

Summarizing our results to this point, we found evidence that both TNF and MTHFR are associated with both depression and alcohol in the literature, TNF and MTHFR interact to influence the comorbidity and alcohol exerts an environmental influence on this genetic network, and folate levels are altered in both depression and alcohol dependence. Investigating the environmental influence of ethanol on TNF, we found that ethanol intake suppresses the function of TNF Alpha Converting Enzyme (TACE, a.k.a. ADAM17, GeneID 6868) [28], leading to reduced TNF signaling. Integrating these lines of evidence, we propose a model of comorbid depression with AUD (Figure 3). In this model, environmental ethanol exposure suppresses TNF signaling by inhibiting TACE [28]. Reduced TNF signaling reduces TNF-R1 activation [30,31], which then reduces NF-kB activation [32]. NF-kB activates the expression of both TNF [33,34] and MTHFR [35,36], so reduced NF-kB activation contributes to further reduction in TNF signaling as well as reduced MTHFR expression. Reduced expression of MTHFR would be expected to alter folate metabolism and increase susceptibility to depression [38].

Signal transduction, leading to transcriptional regulation of both TNF and MTHFR, is an essential assumption of this model. In addition to building networks, GeneGo analyzes networks for over-representation of genes that participate in Gene Ontology (GO) processes (similar to the DAVID analysis described), then ranks the GO processes by p-value. Table 5 shows that "positive regulation of transcription from RNA polymerase II promoter" is the most significantly over-represented GO process in this network. This result is consistent with the hypothesis that environmental influences, via ethanol exposure, are transduced through this genetic network to alter TNF and MTHFR expression, folate metabolism, and susceptibility to depression.

**Table 5:** Ranking of Gene Ontology processes for GeneGo network in Figure 2

**Gene Ontology Process**	**p-value**
positive regulation of transcription from RNA polymerase II promoter	3.8E-06
negative regulation of interleukin-12 biosynthetic process	5.3E-06
inflammatory response	9.5E-06
positive regulation of I-kappaB kinase/NF-kappaB cascade	1.5E-05
positive regulation of transcription, DNA-dependent	1.5E-05
regulation of I-kappaB kinase/NF-kappaB cascade	1.8E-05
positive regulation of transcription	2.9E-05
positive regulation of nucleobase, nucleoside, nucleotide and nucleic acid metabolic process	3.3E-05
regulation of interleukin-12 biosynthetic process	3.8E-05
interleukin-12 biosynthetic process	3.8E-05
response to wounding	3.9E-05
interleukin-12 production	4.8E-05

Regulation of transcription is the most significantly over-represented Gene Ontology process for this network, consistent with the hypothesis that environmental influences, via ethanol exposure, are transduced through this genetic network to alter TNF and MTHFR expression, folate metabolism, and susceptibility to depression.

Apolipoprotein E (APOE) was excluded from the formal analysis because the annotation in Entrez Gene was inferred, rather than curated. In pursuing the hypothesis that APOE is involved in comorbid depression with AUD, we re-started the analysis including APOE. We queried PubMed for citations consistent with this hypothesis (Table 2) and found evidence of APOE's role in both depression and AUD, but no evidence for a role in the comorbidity. PDG-ACE analysis did not produce any significant results after correction for multiple hypothesis tests. We attempted to insert APOE into the GeneGo network (Figure 2) and found no annotated interactions between APOE and the other genes in the network. Apolipoprotein E may well have an influence on comorbid depression with AUD but, if so, the mechanism is either independent of the network modeled or, more likely, the data is not yet available to reliably connect APOE to this network.

## Discussion

Comorbid depression with AUD shows evidence of both genetic and environmental influences on susceptibility. In three phases of hypothesis generation and testing (NCBI database queries, PDG-ACE and GeneGo analyses) we established and tested a model of gene-by-environment interaction that shows evidence of influencing the comorbidity and is consistent with established knowledge about AUD and depression [37]. We first hypothesized that common genetic influences affect AUD and depression. We tested this hypothesis by searching for candidate genes and found published evidence, via Entrez Gene and PubMed, supporting the roles of TNF and MTHFR in depression and AUD. Given evidence of a multi-gene influence on the comorbidity, we hypothesized that TNF and MTHR participate in a genetic interaction influencing the comorbidity. Mining the Entrez Gene records for TNF and MTHFR via the PDG-ACE algorithm, we found twenty one keywords that are common and significantly over-represented across the gene pair, consistent with interaction between these genes in comorbid AUD with depression. In addition, among the significant keywords, those that are most often associated with depression and AUD in the literature are suggestive of an environmental effect on this genetic interaction via ethanol intake or consumption. Given evidence that TNF and MTHFR participate in a genetic interaction that may be influenced by environmental exposure to ethanol, as well as evidence that TNF influences signal transduction pathways in response to the environment, we hypothesized signal transduction as the most appropriate model of the gene-by-environment interaction. Modeling this hypothesis via GeneGo, we found that both TNF and MTHFR are influenced by a genetic feedback cycle that incorporates environmental ethanol exposure into folate metabolism. Altered folate levels, as well as AUD, are consistently linked to depression [42-44].

**Figure 4:**
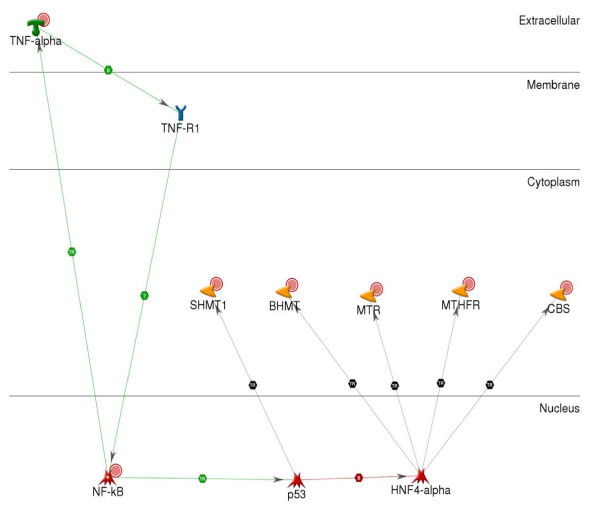
**GeneGo graphic illustrating how TNF signaling impacts one-carbon metabolism**. Folate metabolism is one element of one-carbon metabolism and TNF signaling influences all of the enzymes involved. As a result, variations in any of the genes involved in one-carbon metabolism could influence folate metabolism and susceptibility to depression.

Mason and Choi [37] review other mechanisms (decreased dietary intake of folate, decreased intestinal absorption, increased urinary secretion, and cleavage of the folate molecule) that have been purported to reduce the bio-availability of folate with excessive ethanol intake. In addition, they review the adverse effects that ethanol can have on one-carbon metabolism, a process that includes the synthesis of folate. Mason and Choi show five enzymes involved in one-carbon metabolism including MTHFR, Cystathionine beta-synthase (CBS, GeneID 875), Betaine Homocysteine Methyltransferase (BHMT, GeneID 635), Serine Hydroxymethyltransferase 1 (SHMT, GeneID 6470), and Methionine Synthase (MTR, GeneID 4548). Figure 4 is a GeneGo graphic showing how TNF signaling may impact every one of these enzymes by regulating their expression. This network, still fairly simple, includes all of the elements of the proposed model (Figure 2) and provides a suggestion of how AUD impacts one-carbon metabolism in a complex genotype-phenotype relationship. Arguably, because all of the enzymes seen in this network affect folate metabolism, they all are candidates for influencing comorbid depression with AUD.

Based on the relationship between folate metabolism and one-carbon metabolism, we searched for evidence that the one-carbon metabolic process influences depression, AUD, and the comorbidity. Table 6 shows evidence that several of these genes may have influences on depression or AUD. The larger hypotheses, depression AND folate, ethanol AND folate, show 133 and 237 citations, respectively, though we did not find evidence for depression AND ethanol AND folate. In Figure 4, two additional genes, p53 (TP53, GeneID 7157) and HNF4a (HNF4-alpha, GeneID 3172), are shown to participate in the network. Searching for evidence of these genes in depression, AUD, and the comorbidity, we found evidence for association between p53 and ethanol (Table 6). Interestingly, "p53 gene" is one of the 21 keywords that we found to be common and significantly overrepresented across the TNF/MTHFR gene pair in the PDG-ACE analysis (Table 4).

**Table 6:** Additional hypotheses tested based on GeneGo modelling

**Short form, PubMed Queries**	**# PubMed Citations**
depression AND CBS	1
ethanol AND CBS	2
depression AND ethanol AND CBS	0
depression AND BHMT	0
ethanol AND BHMT	6
depression AND ethanol AND BHMT	0
depression AND SHMT	0
ethanol AND SHMT	0
depression AND ethanol AND SHMT	0
depression AND MTR	1
ethanol AND MTR	17
depression AND ethanol AND MTR	0
depression AND folate	133
ethanol AND folate	237
depression AND ethanol AND folate	0
depression AND p53	0
ethanol AND p53	43
depression AND ethanol AND p53	0
depression AND HNF4a	0
ethanol AND HNF4a	0
depression AND ethanol AND HNF4a	0

Based on GeneGo modelling, additional candidate genes were tested for association with depression, AUD, and the comorbidity on 9 December, 2007. As in Table 2, queries were qualified by Medical Subject Headings (MeSH) annotation to minimize spurious associations. The actual queries, including MeSH qualification, are provided as Additional Materials (Tables_2_and_6_Detailed.xls). Column 1 has a short form of the query and column 2 shows the number of citations that meet the query criteria.

Notably, while the evidence assembled in this analysis is consistent with our hypothesized model of gene-by-environment interaction of comorbid depression with AUD, much data remains missing. For example, we emphasize the environmental influence of alcohol consumption, though we have little information on how alcohol dosage or the duration of alcohol exposure might affect genetic influences in our model. Equally, while we have identified a network of genes that may influence the comorbidity, we have limited information on heritable variation that would be expected to increase or decrease susceptibility. To date, the C677T variant of MTHFR is associated with both susceptibility to depression [27,45,46] and ethanol response [26], and levels of TNF mRNA have been associated with depression [25]. Cytogenetic band 6p21, the chromosomal location of TNF, has recently been associated with chromosomal aberrations in alcoholism [47]. However, the genetic influences of this network are likely to be much more complex than what we know so far. Missing data on these influences await follow-on analyses (e.g., targeted genotyping in an affected population versus controls, animal modelling) that can be informed by the model developed here. Also, given the model proposed, future data from WGA or microarray studies can be tested on a reduced number of hypotheses, thus increasing the power of these tools.

### Implications

Implications of this model are consistent with dietary guidance recommending monitoring and appropriate supplementation of folate for patients in treatment for depression [48-50], AUD, or comorbid depression with AUD [37,38,42-44]. In addition, identification of the variants associated with risk could be useful in prognosis and treatment of either or both conditions [51]. Pharmacogenomic approaches are being successfully implemented in psychiatry, to the great benefit of patients with specific genetic variants [52-54], and the identification of disease predisposing variants will likely improve the prognosis for depression with AUD.

## Conclusion

The model developed in this analysis represents one mechanism whereby, for genetically susceptible individuals, alcohol intake could lead to altered folate metabolism and increased susceptibility to depression. The effect of excessive alcohol consumption on folate levels is not new [37] but this analysis puts the environmental effect of alcohol consumption into a genetic context and provides a model for further hypothesis testing.

Candidate gene approaches offer a necessarily limited view of gene-disease association because so much data is missing on virtually all genes. In addition, data mining approaches, if not carefully controlled, can lead to false positive associations. However, by starting with the most reliable data available to select candidate genes, using multiple lines of evidence to test the validity of candidates, then putting the candidates into context, we believe we have developed one valid model of gene-by-environment interaction influencing comorbid depression with AUD. The proposed gene-by-environment interaction model provides a biologically plausible and testable hypothesis on the genetic etiology of comorbid depression with AUD.

With appropriate caveats, this approach to complex disease analysis could be applied to understanding many diseases. Both publication bias towards positive results and bias towards genes that are commonly studied will tend to steer text based evidence in the direction of a relatively limited set of genes. This bias will be countered by the large volumes of data currently being generated without prior hypotheses. In particular, WGA and microarray data are being deposited in publicly available datasets. These data will complement text based approaches by allowing for unbiased tests of the reduced number of hypotheses posed. Indeed, as the volume of data available in databases increases, this type of analysis will become more valuable.

## Competing interests

The authors declare that they have no competing interests.

## Authors' contributions

RCM initially conceived of the work, conducted the database queries as well as the PDG-ACE and GeneGo analyses, and wrote the first draft of the manuscript. BJK developed the PDG-ACE algorithm and helped in subsequent drafting of the manuscript. EFHS put the initial results in the context of one-carbon metabolism and helped with subsequent drafting of the manuscript. MGM participated in the design and coordination of the study and helped draft the final manuscript. All authors read and approved the final manuscript.

## Supplementary Material

Additional file 1 Detailed PubMed database queries. Click here for file

Additional file 2 Manuscript describing the PDG-ACE algorithm. Click here for file

Additional file 3 List of 2,531 keywords used in PDG-ACE analysis. Click here for file
