# Supplementary material for: Modeling gene-by-environment interaction in comorbid depression with alcohol use disorders via an integrated bioinformatics approach
Source: BioData Min. 2008 Jul 17;1:2. doi: 10.1186/1756-0381-1-2 (PMC2547861; doi:10.1186/1756-0381-1-2)
Supplement: Additional file 2 — Manuscript describing the PDG-ACE algorithm. [file 1756-0381-1-2-S2.doc]

Identifying Hypothetical Genetic Influences on Complex Disease Phenotypes

Benjamin J. Keller, PhD^1,4^; Richard C. McEachin, PhD^2,4^; Richard M. Watanabe, PhD^3,4^; Melvin G. McInnis, MD^2,4^

^1^Eastern Michigan Univ., Ypsilanti, MI; ^2^Univ. of Michigan, Ann Arbor, MI;
^3^Univ. of Southern California, Los Angeles, CA; ^4^National Center for Integrative Biomedical Informatics

Abstract

We describe a heuristic algorithm for generating hypothetical genetic influences from loci associated with a complex disease phenotype. This approach, called Prioritizing Disease Genes by Analysis of Common Elements (PDG-ACE), mines keywords from text descriptions of genes and uses them to relate genes close to disease-associated loci. The keywords common to pairs of gene descriptions may represent preliminary hypotheses about the biological relationship between the genes, and the role the genes play in the disease phenotype. We discuss validation experiments that show that the approach is able to find relationships that have been previously been published, and also experimentation that indicates the approach is robust to differences in keyword vocabulary. We outline a brief case study in which results from a recently published Type 2 Diabetes association study are used to identify potential hypotheses.

Introduction

Identifying, confirming, and validating candidate genes in complex diseases is a daunting task. A genome-wide association study, in particular, can generate an extremely long list of single nucleotide polymorphisms (SNPs) showing association with the phenotype, each of which may impact gene function in a number of ways. While it is common to consider only genes close to a disease-associated SNP, this still implicates a large number of genes that must be filtered. We consider this filtering task as one of finding hypothetical genetic influences on the disease phenotype, which may help explain how the gene is involved in the disease. Our approach is to mine Entrez Gene^1^ records for keywords that are common to the descriptions of genes near pairs of disease-related loci. In cases where the keyword occurs in a consistent context in the descriptions of both genes, the keyword may lead to a novel hypothesis on the genetic etiology of the disease phenotype.

While similar strategies are used by other candidate gene finding tools^2^, the use of keywords to mine unstructured gene descriptions is novel. The dual approach to ours is to look for co-occurrence of genes in the literature, as is done by PDQ Wizard^3^. While it is also possible to find hypothetical genetic influences using this approach, genes may co-occur in a paper for many reasons, most of which will not provide useful annotation of the gene.

This paper describes our strategy and its implementation in a tool called PDG-ACE (Prioritizing Disease Genes by Analysis of Common Elements). We first describe the mining of the Entrez Gene records, followed by the algorithm and statistical tests. Then we describe experimentation for validation and parameter tuning. We present a case study using the genes identified in a recent Type 2 Diabetes (T2D) study^4^. And, finally, we end with a discussion.

Mining Gene Descriptions

The PDG-ACE algorithm uses an association of keywords with genes mined from Entrez Gene records. We have developed tools that build these associations (essentially deriving keyword vocabularies) in two ways: matching Entrez Gene description text against a dictionary of keywords, and naïve recognition of phrases within the descriptions. The first assumes a dictionary of keywords, which may be phrases of words separated by underscore characters, and finds all longest full matches against the dictionary in the text. The second finds the longest non-stopword phrases within the text. In both cases, stopwords are filtered out, using a stopword list consisting of common English words.

For experimentation, we constructed three vocabularies. In each case, we first derived an initial vocabulary, and then filtered the keywords to keep only those keywords likely to be common and over-represented in Entrez Gene records. The first vocabulary is based on Medical Subject Headings (MeSH), from which we created a vocabulary by splitting headings to make phrases more likely to be seen in text. We created the second vocabulary, meant to eliminate bias from using a particular dictionary, by extracting naïve keyphrases directly from the Entrez Gene records. The third vocabulary was created to emphasize keywords related to neurological disorders. To do this, we extracted naïve keyphrases from OMIM^5^ records containing the substring “neuro”, and used these as the dictionary. Below, we refer to these as MeSH, NAÏVE and OMIM vocabularies.

Once the initial association is mined, we screen the vocabulary to eliminate keywords that are very rare or very common in Entrez Gene records. We apply an iterative refinement based on an approximation to the statistical significance test used in the algorithm. If we consider the occurrence of a keyword in a pair of gene descriptions, we can define the *p*-value as the probability that the keyword occurs in randomly selected pairs of genes. Letting *G* be the total number of genes, and *N* be the total number of keywords, and assuming a Bonferroni correction of 0.05/*N*, we want keywords with at most (0.05*G*^2^/*N*)^1/2^. We eliminate keywords with fewer than three occurrences. This refinement process narrows the vocabulary to words that are likely to be common across gene pairs and pass the significance test for over-representation.

Note that in preprocessing we build a list of genes and their locations from an authoritative source. Results presented here are based on hg18 data tables from the UCSC genome browser^6^. Filtering of genes may occur first when finding the correspondence between RefSeq accession numbers from UCSC tables and Entrez Gene IDs. More filtering occurs because we mine only Entrez Gene records marked as ‘live’.

Algorithm

The primary input to PDG-ACE is a pair of disease-associated loci and a delta in base pairs from each locus. These inputs define a pair of chromosomal regions from which genes are considered. The algorithm does one run using this observed pair of disease-associated regions, and then performs permutation runs to determine the significance of the observed results.

In a run, each keyword is scored with the product of the number of genes from each region whose description contains the keyword. The score is the number of possible pairs of genes across the regions that the keyword represents. All keywords common to at least one gene in each region will have a nonzero score.

The observation run assigns a score to each keyword, and keywords that have a zero score are filtered out prior to the permutation runs. The permutations are run on blocks consisting of the same number of sequential genes as in the observed regions. A block is selected by first randomly choosing a chromosome arm, and if possible, randomly picking a block of sequential genes on that chromosome arm. If the arm is too small, then another arm is chosen until one that has enough genes is found.

As permutations are run, the rank of each observed keyword score is determined. If, on completion of the permutation runs, the score of a keyword ranks above a user given threshold, then the keyword, its rank, and the corresponding genes from both regions are reported. The *p*-value for a keyword is calculated as the proportion of scores for permutation runs that are greater than or equal to the observation run score. A Bonferroni correction is then applied so that the threshold for significant *p*-values is 0.05/*N*, where *N* is the total number of keywords in the vocabulary.

Because we are using permutation testing, some trials are needed to find the number of permutations at which the sample of the genome is sufficient to yield consistent significance for high-scoring keywords. When using PDG-ACE, we start with a minimum of one million iterations and run each test in duplicate. We consider the sample to be sufficient if the top three keywords are identical, and in the same order in the two samples. If that sampling sufficiency criterion is not met, we increase the number of permutations and re-run the test in duplicate until the criterion is met.

Experimentation

We validated our approach using published studies as positive controls, and randomly selected gene pairs as negative controls. Most of the control studies used genes that correspond to SNPs as loci, but we also ran experiments using two other studies that used microsatellite markers as loci. We ran experiments with all three of the vocabularies described above.

For validation, the positive controls were based on seven published studies showing statistically significant gene-gene interactions. These include two breast cancer studies^7, 8^, as well as osteoporosis^9^, anorexia nervosa^10^, colorectal cancer^11^, asthma^12^, and neural tube defect studies^13^. Each of these studies found statistical evidence of gene-gene interactions. Our expectation was that PDG-ACE would find over-represented common keywords consistent with genetic interactions predisposing these diseases. The negative controls were pairs of randomly selected genes from Entrez Gene, with the expectation being that PDG-ACE would not find over-represented common keywords.

For each locus pair, we tested regions defined by deltas from 1 kilobase pairs (KBP) to 10 megabase pairs (MBP) from each gene's Transcription Start Site (TSS). At each delta, we ran the PDG-ACE algorithm in duplicate and performed enough permutations to ensure a sufficient sample. Each test was performed in parallel, using all three vocabularies (OMIM, MeSH, and NAIVE). In all but one case, the results for deltas at 1 MBP and above showed no significant common keywords; we report only results for smaller regions.


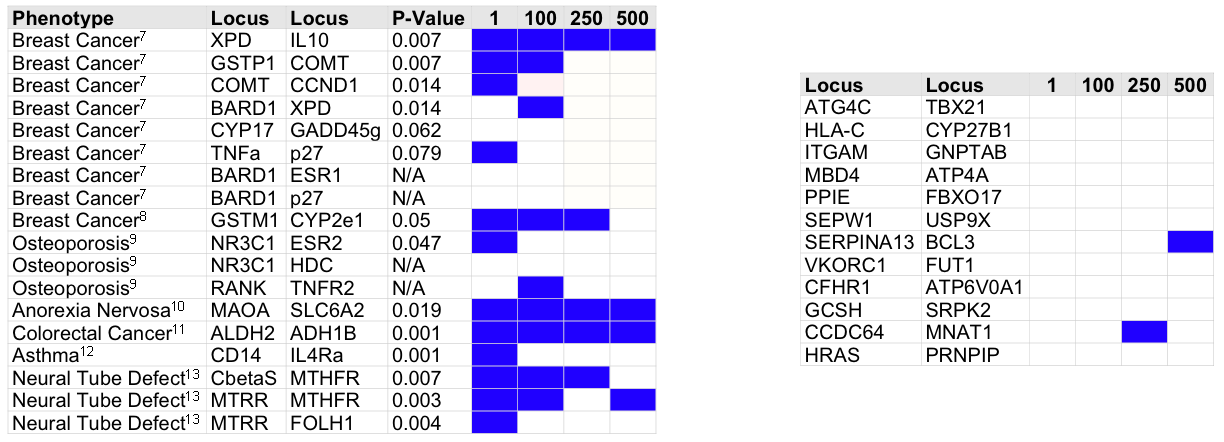


Table 1. Results of validation experiments showing hits in the positive (left) and negative (right) controls.

The *p*-values are from the original study, and numbers refer to the delta from the loci in KBP.

Table 1 shows the hits for the positive and negative controls, using the MeSH vocabulary of 2531 keywords. Note that the overall pattern of hits in the positive controls is significantly different from that seen in the negative controls (chi-square p-value < 0.01). In general, the strongest evidence for multi-gene effects is near the observed loci (1 KBP delta), and the pattern of hits is generally consistent with the *p*-values found in the control studies. As expected, in most, but not all, cases the significantly over-represented, common keywords are consistent with the disease etiology. For example, in the first breast cancer study, the COMT-CCND1 genetic interaction is significant (*p*-value 0.014 in the interaction study) and the over-represented, common keyword is “estradiol” (*p*-value 0.041 after a Bonferroni correction for 2531 hypothesis tests). The keyword “estradiol” is used in the same context at both loci in the locus pair, and may offer insight into hormone sensitive breast cancer etiology.

In two cases, gene families provide the strongest evidence at a given locus pair. For the BARD1-XPD (a.k.a. ERCC2) interaction in the first breast cancer study (corrected *p*-value 0.014), BARD1 as well as paralogs ERCC2 and ERCC1 all refer to the keyword “dna repair” (corrected *p*-value 0.009). Since ERCC2 and ERCC1 are adjacent in the genome, evidence of the multi-gene effect extends beyond the bounds of the XPD gene, out to +/-100 KBP. Arguably, any cancer-related effects of variations in ERCC2 may be influenced by variations in ERCC1, so both of the ERCC genes should be evaluated for genetic variation related to breast cancer. A similar effect is seen for the RANK (a.k.a. TNFRSF11)-TNFR2 (a.k.a. TNFRSF1B) interaction in the osteoporosis study, where TNFRSF1B and TNFRSF8 are adjacent in the genome. In this case, the authors of the previous study did not find significant evidence for a genetic interaction. However, all three genes refer to keyword “marrow” (corrected *p*-value 0.033), consistent with bone disease, so the true genetic interaction may have been hidden in the previous study but revealed by PDG-ACE. In both the breast cancer and osteoporosis studies, the evidence is consistent with gene family effects on the phenotype, as expected in complex diseases.

These validation experiments show that the findings from PDG-ACE are generally consistent with the strength of prior evidence, as seen by comparing the *p*-values found in the interaction analyses and the pattern of common, over-represented, keywords found by PDG-ACE. In general, evidence of commonality falls off as the interval grows larger. Negative controls generally show no evidence of common effects, as expected for randomly selected gene pairs (Table 1).


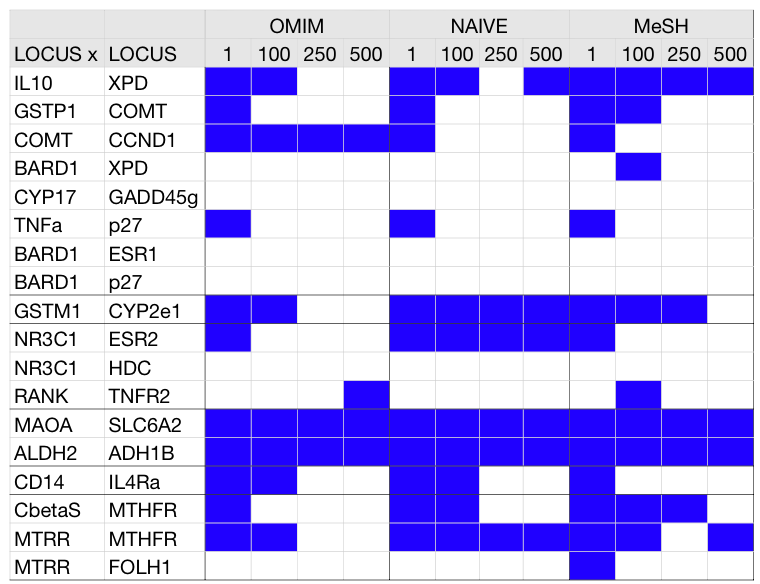


Table 2. Hits for OMIM, NAÏVE and MeSH vocabularies.

We tested PDG-ACE on two additional genetic interactions studies^14, 15^, each based on variation in microsatellite markers, which would be a common input to PDG-ACE from a genetic linkage study. Results of these experiments (not shown) indicate that PDG-ACE is not effective for this type of prior information and is best used to look at the direct gene-gene relationships available from a genome-wide association or gene-gene interaction study.

Finally, we did experiments to study the impact of choosing a particular vocabulary by repeating the positive control experiments using each of the three vocabularies (MeSH, OMIM, and NAÏVE). We ran the experiments in triplicate, using identical parameter settings for each of the vocabularies. Table 2 shows the results from these experiments. Interestingly, the pattern of hits is quite similar for all three vocabularies, even though the specific keywords in the vocabularies are different. For example, for the GSTM1-CYP2e1 locus pair at 1 KBP in the second breast cancer study, the common over-represented keywords for the MeSH vocabulary are: “cyp2e1”, “ethanol”, “smoke”, “area”, “stomach”, “toxicity”, and “xenobiotics”. For the NAÏVE vocabulary the corresponding list is: “alcoholics”, “cigarette smoke”, “high-risk area”, “stomach cancer”, “incomplete intestinal metaplasia”, “non-small cell lung carcinoma”, and “pancreatitis”. For the OMIM vocabulary, the keywords are: “workers”, “metabolizing”, and “increased susceptibility”. We speculate that if there are any relevant biomedical keywords in common between two gene descriptions, then there are likely to be other keywords in common. Our conclusion from these experiments is that PDG-ACE is relatively robust to the vocabulary is used.


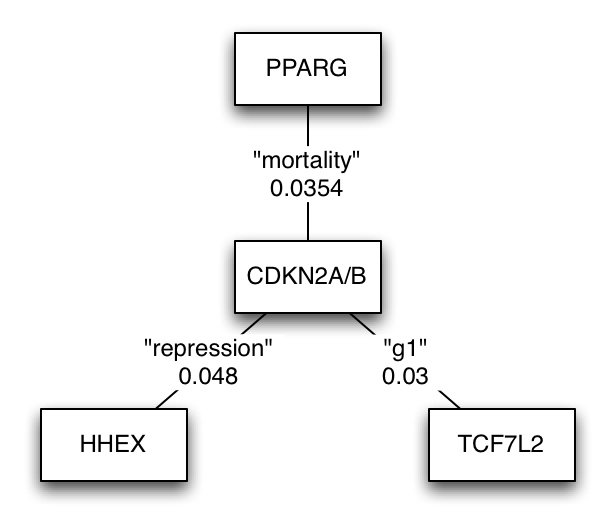


Figure 1. PDG-ACE discovered relationships between CDKN2A/B and known T2DM genes in the FUSION study. Edge labels are keywords, and their *p*-values.

Case Study: Type 2 Diabetes Mellitus

As an example of how PDG-ACE can aid in the understanding of complex disease etiology, we discuss its application. A recently published study^4^ identified ten T2D-associated loci; five corresponding to genes previously associated with T2D, and five which had no prior association with T2D. Two of the loci are excluded, because one (rs9300039) is more than 1 MBP from the nearest annotated gene, and the other (rs8050136) is near the FTO gene, which is annotated as provisional in Entrez Gene and so was excluded by PDG-ACE. Using the remaining T2D-associated genes as input (IGF2BP2, CDKAL1, CDKN2A/CDKN2B, PPARG, SLC30A8, HHEX, TCF7L2, KCNJ11) we ran PDG-ACE with the MeSH vocabulary. We performed at least one million iterations for each test, and confirmed that each sample was sufficient, as described above. We searched up to +/-500 KBP from the TSS for each locus.

As shown in Figure 1, PDG-ACE found significant commonality between the CDKN2A/CDKN2B gene pair and three other T2D candidate genes. No significant multi-gene effects were found for the PPARG-HHEX, PPARG-TCF7L2, and HHEX-TCF7L2 locus pairs. Notably, the CDKN2A/B locus was newly discovered by Scott, *et* *al*.^4^, while all three of the genes related to CDKN2A/B by PDG-ACE were previously established as T2DM candidates.

The observation that the CDKN2A/B gene pair shows significant multi-gene effects with all three of these other T2D associated genes led us to the hypothesis that these genes form a cluster that may participate in a larger multi-gene effect that could be related to T2D susceptibility. To test this hypothesis, we used GeneGo^16^ to assess over-representation of the PDG-ACE identified gene set in Gene Ontology (GO) processes. Parameter settings used in GeneGo's “analyze networks” algorithm were to use only curated interactions, where the interactions included binding, direct/indirect, or unspecified types. GeneGo separates CDKN2A transcripts into two isoforms, p14ARF and p16INK4, yielding six entities. GeneGo finds that all six entities fit into the GO process “regulation of cellular process”, GO:0050794, and the input set is significantly over-represented in this process, with a *p*-value <0.01. Notably, the keywords found by PDG-ACE that relate the genes in the cluster are all consistent with the regulation of cellular processes.

Discussion

The PDG-ACE algorithm takes a simplified approach to complex disease analysis. Assuming that multiple genetic influences converge on a single phenotype in complex diseases, we search for common elements of text describing genes at disease-related loci, revealing potential underlying genetic influences on the phenotype of interest. Existing tools look for common elements of annotation among multiple genes including pathways, gene ontology, and expression. However, for most genes the annotation of these details is incomplete. The heuristic employed in PDG-ACE overcomes this shortcoming by using available text descriptions for genes, and is promising for generating hypotheses for genetic influences on complex disease. Clearly, however, PDG-ACE implements only an initial step in the refinement of such hypotheses; other existing tools complement the approach.

**Acknowledgements**

We wish to thank Mohsen Almani, Glenn Tarcea, Usha Reddi, and Pratima Naik for programming assistance. This work was partially funded by NIH Grant # U54-DA-021519, and the Prechter Bipolar Research Fund (RCM and MGM).

References

1. Maglott D, Ostell J, Pruitt KD, Tatusova T. Entrez Gene: gene-centered information at NCBI, Nucleic Acids Res. 2005;33:D54-D58.
2. Oti M, Brunner HG. The modular nature of genetic diseases. Clin Genet. 2007;71:1–11.
3. Grimes GR, Wen TQ, Mewissen M *et.al.* PDQ Wizard: automated prioritization and characterization of gene and protein lists using biomedical literature. Bioinformatics 2006;22(16):2055-7.
4. Scott LJ, Mohlke KL, Bonnycastle LL *et al*. Genome-wide association study of type 2 diabetes in Finns detects multiple susceptibility variants. Science. 2007;316(5829):1341-5.
5. Hamosh A, Scott AF, Amberger JS *et al.* Online mendelian inheritance in man (OMIM), a knowledgebase of human genes and genetic disorders. Nucleic Acids Res. 2005;33:D514-D517.
6. Kent WJ, Sugnet CW, Furey TS, Roskin KM, Pringle TH, Zahler AM, Haussler D. The human genome browser at UCSC. Genome Res. 2002;12(6):996-1006.
7. Onay VU, Briollais L, Knight JA *et al.* SNP-SNP interactions in breast cancer susceptibility. BMC Cancer. 2006;6:114.
8. Wu SH, Tsai SM, Hou MF *et al*., Interaction of genetic polymorphisms in cytochrome P450 2E1 and glutathione S-transferase M1 to breast cancer in Taiwanese woman without smoking and drinking habits. Breast Cancer Res Treat. 2006;100(1):93-8.
9. Xiong DH, Shen H, Zhao LJ *et al*., Robust and comprehensive analysis of 20 osteoporosis candidate genes by very high-density single-nucleotide polymorphism screen among 405 white nuclear families identified significant association and gene-gene interaction. J Bone Miner Res. 2006;21(11):1678-95.
10. Urwin RE, Bennetts BH, Wilcken B *et al.* Gene-gene interaction between the monoamine oxidase A gene and solute carrier family 6 (neurotransmitter transporter, noradrenalin) member 2 gene in anorexia nervosa (restrictive subtype). Eur J Hum Genet. 2003;11(12):945-50.
11. Matsuo K, Wakai K, Hirose K *et al*., A gene-gene interaction between ALDH2 Glu487Lys and ADH2 His47Arg polymorphisms regarding the risk of colorectal cancer in Japan. Carcinogenesis. 2006;27(5):1018-23.
12. Lee S, Kim H, Kim J, Kim B, Kang M, Hong S. Gene-gene interaction between CD14 and IL-4Ra polymorphisms is associated with asthma susceptibility in Korean children with asthma. J. Allergy and Clin Immunol. 2006;117(2):S199.
13. Relton CL, Wilding CS, Pearce MS *et al*. Gene-gene interaction in folate-related genes and risk of neural tube defects in a UK population. J Med Genet. 2004;41(4):256-60.
14. Cox NJ, Frigge M, Nicolae DL *et al*. Loci on chromosomes 2 (NIDDM1) and 15 interact to increase susceptibility to diabetes in Mexican Americans. Nat Genet. 1999;21(2):213-5.
15. Chang BL, Lange EM, Dimitrov L *et al*. Two-locus genome-wide linkage scan for prostate cancer susceptibility genes with an interaction effect. Hum Genet. 2006;118(6):716-24.
16. Ekins S, Bugrim A, Brovold L *et al*. Algorithms for network analysis in systems-ADME/Tox using the MetaCore and MetaDrug platforms. Xenobiotica. 2006;36(10-11):877-901.
